# Chemical profile, antimicrobial activity, and leaf anatomy of *Adenophyllum porophyllum* var. *cancellatum*

**DOI:** 10.3389/fphar.2022.981959

**Published:** 2022-10-11

**Authors:** Silvia Aguilar-Rodríguez, Ma. Edith López-Villafranco, María Patricia Jácquez-Ríos, Claudia Tzasna Hernández-Delgado, María Fernanda Mata-Pimentel, Edgar Antonio Estrella-Parra, Adriana Montserrat Espinosa-González, Erick Nolasco-Ontiveros, José Guillermo Avila-Acevedo, Ana María García-Bores

**Affiliations:** ^1^ Laboratory of Botany, UMF, FES-Iztacala, National Autonomous University of Mexico, Mexico City, Mexico; ^2^ Herbarium IZTA, FES-Iztacala, National Autonomous University of Mexico, Mexico City, Mexico; ^3^ Laboratory of Bioactivity of Natural Products, UBIPRO, FES-Iztacala, National Autonomous University of Mexico, Mexico City, Mexico; ^4^ Laboratory of Phytochemistry, UBIPRO, FES-Iztacala, National Autonomous University of Mexico, Mexico City, Mexico

**Keywords:** *Adenophyllum porophyllum* var. *cancellatum*, antifungal activity, aromatic plant, *Candida*, oil gland, oxidized monoterpenes

## Abstract

*Adenophyllum porophyllum* var. *cancellatum*, known as “árnica del monte” in Mexico, is an aromatic annual plant belonging to the Asteraceae family that grows from southern Arizona to central Mexico. The aerial parts of the plant are used in traditional medicine to treat skin diseases such as irritations, infections, and wounds. In this study, the essential oil of this plant was characterized, and its antimicrobial activity was evaluated. This species has large glands in its leaves; therefore, for quality control purposes, an anatomical study of the leaves was performed. The essential oil was isolated from the aerial parts of the plant through hydro-distillation and analyzed using a gas chromatography/mass spectrometry (GC/MS) system. Its anti-yeast activity was evaluated against three *Candida* species and ten bacterial strains using the disk diffusion technique. The minimum inhibitory concentration (MIC), minimum fungicidal concentration (MFC), and minimum bactericidal concentration (MBC) were determined using broth microdilution. Anatomical study was performed on the middle part of the leaf. A yield of 0.5% of the essential oil was obtained from the herb, and Eighteen compounds in the essential oil were identified, within them *trans* pinocamphone (29.5%), limonene (24.7%), pinocarvone (21.8%), and *cis* pinocamphone (8.0%) were the main components. The inhibition zones were between 10 mm and 20 mm, and the MIC and MFC against the three *Candida* species ranged from 60 to 500 μg/ml. The leaf anatomy showed anisocytic stomata, simple and glandular trichomes of different types, and large and elliptical-shaped lysigenous glands, which can be used for taxonomic identification. The *A. porophyllum* var. *cancellatum* essential oil can serve as an alternative source of natural antimicrobial agents as an affordable approach to control infectious diseases. This is the first study that reports the chemical composition and antimicrobial activity of the essential oil, as well as the leaf anatomy of this species.

## Introduction

Antimicrobial agents are essential to reduce the global burden of infectious diseases (Manandhar et al., 2019). Fungal and bacterial pathogens affect over a billion people worldwide, particularly those causing skin, nail, and hair infections (Siscar-Lewin et al., 2022). Infectious diseases are complicated by the presence of drug-resistant microorganisms (Sharma et al., 2017). The World Health Organization (2017) published a list of bacterial pathogens for which new antimicrobial developments are urgently needed. Within this broad list, ESKAPE (*Enterococcus faecium*, *Staphylococcus aureus*, *Klebsiella pneumoniae*, *Acinetobacter baumannii*, *Pseudomonas aeruginosa*, and *Enterobacter* species) pathogens were designated as priority. *Candida* spp., are among the most common human pathogenic fungi (Kölher et al., 2017), which cause a mortality of at least 50% in hospitalized patients (De Moraes, 2022). There is an urgent need for the development of new antimicrobial drugs due to increase in the incidence of antibiotic-resistant infections (Wu et al., 2019).

Plant secondary metabolites, which are widely used to treat various infectious diseases, are known for their unique chemical diversity and bioactivity (Soliman et al., 2017). The World Health Organization estimates that 80% of the world’s population relies on traditional medicine based on plant remedies. The study of medicinal plants has led to the discovery of compounds that can be used to modify existing drugs or design new therapeutic alternatives (Rolnik and Olas, 2021). Traditional medicine has enabled the discovery of plant metabolites with therapeutic properties against pathogens associated with human diseases (Lerato et al., 2021). The Asteraceae family is extensively used in traditional medicine and is one of the largest botanical families. Its members have several morphological characteristics in common and a wide variety of specialized metabolites. This family is a very important source of medicinal species with several bioactive compounds, such as terpenes from essential oils that have great potential as antimicrobial agents (Kostić et al., 2020). However, owing to the demand for natural products in international trade, the possibility of adulteration of plant species has been increasing. This has resulted in various adverse consequences; adulteration has put the health and safety of consumers at risk. Currently, there are no standard protocols or practices for identifying and evaluating the different plant species or parts used in natural herbal products (Srirama et al., 2017). Different tools have been used to obtain information that helps in the authentication of plant samples, among them are chemical markers and microscopic characteristics (Ramzan et al., 2019). In the case of the anatomical aspects of Asteraceae, several reports using different approaches are published (Rivera et al., 2019). Some of these investigations describe species used in traditional medicine; for example, Younis et al. (2020) characterized the epidermal attributes of 20 medicinally important species in Asteroideae.

The genus *Adenophyllum* belongs to the Asteraceae family and includes 12 species; some of their traditional use, chemical composition, and biological properties have been evaluated. *A. appendiculatum* (Lag.) Strother (= *Dyssodia appendiculata* Lag.) is used in Oaxaca, Mexico to treat candidiasis and pain (head, stomach, and gums) and has antibacterial activity (Frei et al., 1998; Cilia-López et al., 2021). *A. aurantium* (L.) Strother is used as an infusion to treat intestinal diseases (amoebiasis). The ethyl acetate root extract is effective against *Entamoeba histolytica* trophozoites and to prevent different steps of the parasite’s pathogenic process, including encystment, liver abscess development, fibronectin adhesion, and erythrophagocytosis. This effect may be due to the action of thiophenes such as α-terthienyl and 5-(4″-hydroxy-1″-butynyl)-2-2′-bithiophene, which are the main components of the extract (Herrera-Martínez et al., 2016). In addition, *A. aurantium* roots exhibit activity against phytopathogenic mycelial fungi (Lira-De León et al., 2014), and the aerial parts and roots are nematocidal herbal resources (*Nacobbus aberrans*) (Velasco-Azorsa et al., 2021). However, there are no studies on the anatomical characteristics of the vegetative organs of any of the species in the genus *Adenophyllum*.

*Adenophyllum porophyllum* var. *cancellatum* (Cass.) Strother is the accepted name for an infraspecific taxon of the species *A. porophyllum* (Cav.) Hemsl (Tropicos, 1982). It is located within the subfamily Asteroideae and the tribe Tageteae (Loockerman et al., 2003; Villarreal, 2003; Villaseñor, 2018). *A. porophyllum* var. *cancellatum* is an aromatic annual herb that grows from southern Arizona to central Mexico. This herb is known as “árnica del monte, alcanfor, cardo santo del monte, cimpasúchil, and coronilla” (Villarreal, 2003). The inhabitants from Tonatico, Estado de México, use the aerial organs of the plant (stems with leaves), with or without flowers, to treat skin affections, such as irritation, infections, wounds, and ulcers. The plant is applied topically to the skin in the form of plasters, poultices, and washes. There is only one report on the activity of this species against fungi of agricultural interest (Hernández-Ceja et al., 2021). However, there are no scientific publications that validate the traditional use of this herb or determine its chemical composition or anatomical features.

The objective of this study was to characterize chemically the essential oil, evaluate its activity against *Candida* and bacteria, and conduct an anatomical study of plant leaf to contribute to the pharmacognostic knowledge of *A*. *porophyllum* var. *cancellatum*, and the development of novel alternatives for the treatment of infectious diseases.

## Materials and methods

### Plant material and isolation of essential oil

**FIGURE 1:**
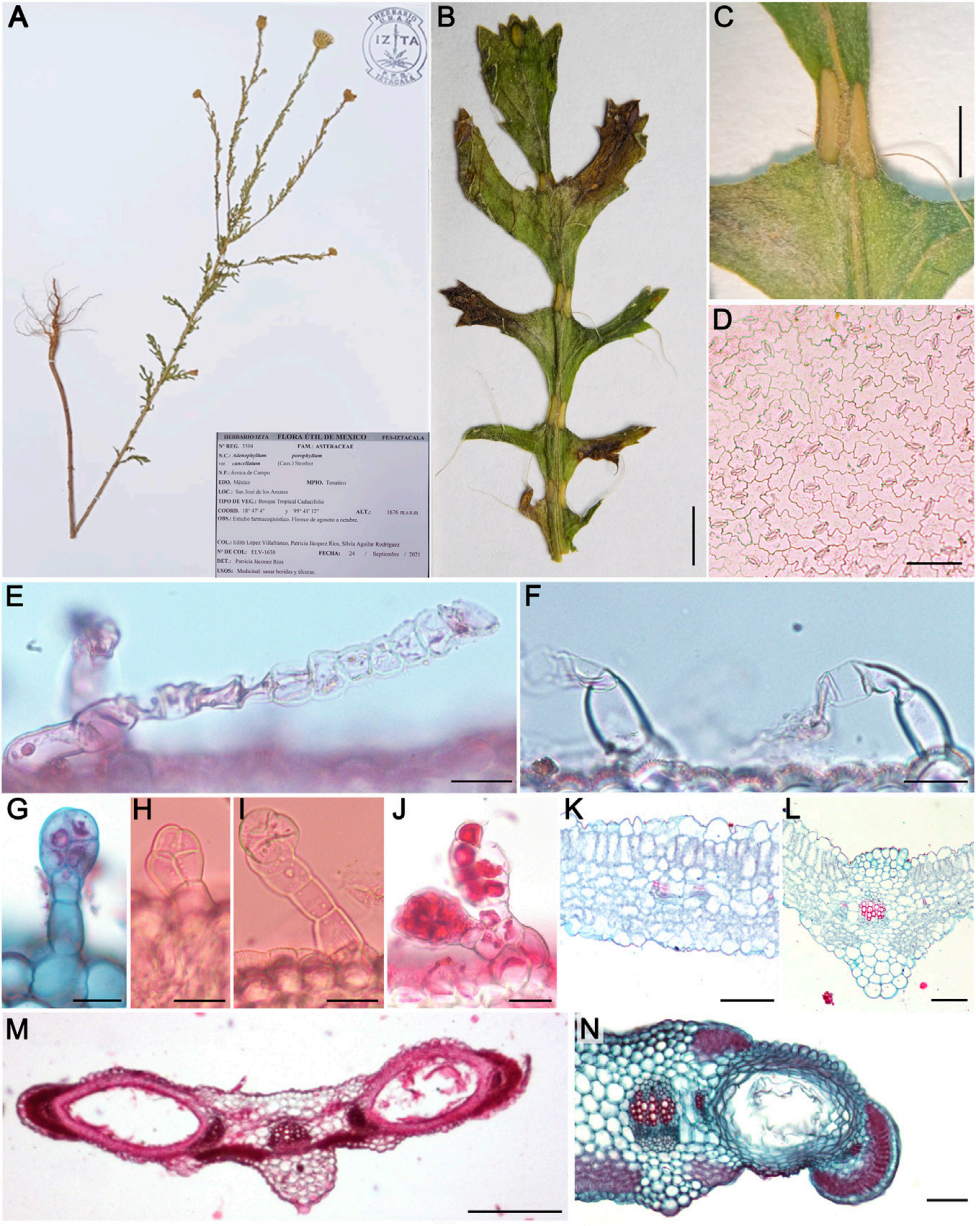
Morpho-anatomical characters of *Adenophyllum porophyllum* var. *cancellatum*: Herbarium specimen No. 3504-IZTA **(A)**, leaf morphology **(B)**, elliptical glands **(C)**, superficial view of abaxial epidermis **(D)**, uniseriate simple trichomes **(E,F)**, glandular trichomes **(G–J)**, transverse view of leaf anatomy (**K**,**L**), glands anatomy (**M**,**N**). Bars: **(B)** = 3 mm, **(C)** = 1 mm, (**D,K,L,N**) = 100 μm, **(E−J)** = 25 μm, **(M)** = 250 µm.

The aerial parts of *A. porophyllum* var. *cancellatum* were collected from San José de los Amates, Tonatico, State of Mexico (September 2021). This zone is located at 18°47′4″ NL and 99°41′12″ WL, at 1677 masl. The vegetation corresponds to a deciduous tropical forest and forms part of the Balsas River Basin. Herborized samples of this species were identified and registered in the ethnobotanical collection of the Herbarium IZTA, Facultad de Estudios Superiores Iztacala, with Registration No. 3504-IZTA (Figure 1A).

A total of 1,500 g of fresh aerial parts were collected, and the essential oil was obtained once through hydro-distillation using a Clevenger-type apparatus. The plant material was heated in water for 3 h in a mantle (6 L), and the steam/oil mix was condensed and collected in a 50 ml funnel. The oil layer was separated and dried over anhydrous sodium sulfate. The isolated essential oil was stored in the dark in a hermetically sealed glass container at −13°C prior to analysis. The yield was calculated as the ratio of the weight of the essential oil obtained to the weight of the plant used (w/w) x100.

### Gas chromatography/mass spectrometry analysis of the essential oil

The essential oil of *A. porophyllum* var. *cancellatum* was analyzed through GC/MS using an Agilent Technologies 7890B Gas Chromatograph (GC) coupled to Agilent Technologies 5977D Mass Spectrometry (MS) Detector under the following conditions: a HP-5MS capillary column (30 m, 0.25 mm, 0.25 μm film thickness), helium (99.999%) as the carrier gas at a constant flow of 1.0 ml/min, an injection volume of 1 μl, an injector temperature of 280°C, and an ion source temperature of 200°C. The oven temperature was programmed to increase from 40°C to 300°C at a rate of 8°C/min, and was then held at 300°C for 5 min. A mass detector was operated in electron impact mode at an ionization energy of 70 eV, with a mass range of 30–600 (m/z).

The oil components were identified through comparative analysis of their retention indices (RI) and mass spectra. The RI values were calculated through linear interpolation relative to the retention times of a series of n-alkanes (C_8_−C_20_ alkane standards, Sigma–Aldrich). Then, the RIs and mass spectra were compared against values in the NIST14 and Mass Spectral Library 15 (National Institute of Standards and Technology, 2022 WebBook, Match > 90%) and literature (Adams, 2007; García-Roja et al., 2009; Lei et al., 2018). The compound concentrations (as % content) were calculated by integrating the corresponding chromatographic peak areas. α -pinene, β-pinene, and *trans*-farnesol were used as standards to verify the correct functioning of the CG/MS system.

### Antimicrobial activity

The anti-*Candida* activity of the essential oil of *A. porophyllum* var. *cancellatum* was evaluated using three strains isolated from clinical cases: *C. albicans* 17MR (donated by the Clinical Analysis Laboratory of FES Iztacala, UNAM); *C. glabrata*, and *C. tropicalis* (isolated from a clinical case and donated by Hospital Angeles Metropolitano, México). The stock culture was maintained on potato dextrose agar (PDA Bioxon) and subcultured twice prior to performing bioassays. Antibacterial activity tests were performed using the following strains: gram-positive bacteria, *Micrococcus luteus* ATCC 10240, *S. aureus* ATCC 29213, *S. aureus* cc, and *S. aureus* CUSI (donated by the Clinical Analysis Laboratory of FES-Iztacala), and *S. epidermidis* FES-C (donated by the Microbiology Laboratory of FES-Cuautitlán), isolated from clinical cases; gram-negative bacteria, *K. pneumoniae* ATCC 13884, *K. oxytoca* ATCC 8724, *Serratia marcescens* ATCC 14756*, Salmonella typhi* ATCC 19430, *Escherichia coli* 82MR, and *E. coli* CUSI (donated by the Clinical Analysis Laboratory of FES-Iztacala), isolated from clinical cases. These strains were maintained at 4°C on Mueller Hinton agar (Bioxon), subjected to sensitivity tests, and sub-cultured twice, before and after the bioassays were performed.

The antifungal and antibacterial activities of the essential oil were evaluated according to the M100 guidelines of the Clinical and Laboratory Standard Institute (CLSI, 2020). A diffusion test was performed using 5 μl (3.25 mg/per disk) of essential oil. Nystatin (50 μg/ml) and chloramphenicol (25 μg/ml) were used as positive controls. The minimum inhibitory concentration (MIC), minimum fungicidal concentration (MFC), and minimum bactericidal concentration (MBC) were determined using microdilution tests with serial dilutions ranging from 3 to 0.06 mg/ml. The experiments were performed in triplicates.

The antibiofilm activity of the *A. porophyllum* var. *cancellatum* essential oil was evaluated using the crystal violet method described by Gómez-Sequeda et al. (2020). Due to the sensitivity to the oil and the fact that they were strains isolated from clinical cases, *C. albicans* 17MR, *S. aureus* cc, and *E. coli* 82MR were used to evaluate the antibiofilm effect. The MIC, MFC, or MBC values were used in all cases. The experiments were performed in triplicates. The percentage biofilm inhibition was calculated as follows:

$$\%Antibiofilm=\frac{OD\;control-OD\;experimental}{OD\;control}\;x\;100$$

OD control: optical density without essential oil; OD experimental: optical density with essential oil.

Three independent experiments were performed for all tests, and the mean and standard deviation of the results were reported. The antibiofilm activity results were analyzed using one-way analysis of variance (ANOVA), where values of *p* < 0.05 were considered statistically significant. In addition, the data were analyzed using Tukey’s test at 95% confidence. Statistical analysis was performed on the collected data using Excel software.

### Anatomical study of the leaf of *A. porophyllum* var. *cancellatum*

An anatomical study was performed on the middle part of the leaf blade, including both wide and narrow areas, and floral involucre bracts. Free-hand sectioning was carried out, and some tissues were cleared with 20% sodium hydroxide and 50% sodium hypochlorite, stained, and mounted in glycerin jelly with safranin (Aguilar-Rodríguez, 1998). Other samples were processed using the paraffin inclusion method (Ruzin, 1999); 15-µm thick cross-sections were obtained using a rotary microtome. Tissue staining was performed using safranin-fast green (Johansen, 1940). Finally, the sections were mounted in a synthetic resin. Images and measurements were obtained using a Nikon E200 microscope attached to an image analyzer (NIS-Elements BR5.21.01; Nikon Instruments). The anatomical descriptions were made according to Metcalfe and Chalk (1979) and Fahn (1985). The bars and brightness of the images were improved using Adobe Photoshop CC 2020.

## Results

### Yield and gas chromatography/mass spectrometry analysis of the essential oil

The yield of essential oil from the aerial parts of *A. porophyllum* var. *cancellatum* was 0.5% (7.5 g); the oil density was 0.7 g/ml. Eighteen compounds were identified in the essential oil using GC/MS analysis. The main components were *trans*-pinocamphone (29.5%), limonene (24.7%), pinocarvone (21.9%), and *cis*-pinocamphone (8.0%) (Supplementary Figure S1). The essential oil of the plant contained only hydrocarbons (31.8%) and oxygenated monoterpenoids (62.1%) but did not sesquiterpenes (Table 1).

**TABLE 1:** The chemical composition of the essential oil of *A. porophyllum* var. *cancellatum*.

No.	Compound	RI _exp_	RI lit	Percentage (%)
1	α-Pinene	938	939–940^a^ ^,^ ^b^ ^,^ ^c^	0.1
2	Sabinene	981	980^a^	1.0
3	β-Pinene	983	979–980^a^ ^,^ ^b^ ^,^ ^c^	1.3
4	β-Myrcene	991	990–993^a^ ^,^ ^b^ ^,^ ^c^	0.4
5	Cymene	1,027	1,024–1,026^a^ ^,^ ^b^ ^,^ ^c^	4.1
6	Limonene	1,031	1,029^a^ ^,^ ^b^ ^,^ ^c^	24.7
7	Eucalyptol	1,034	1,031–1,033^a^ ^,^ ^b^ ^,^ ^c^	1.5
8	γ-Terpinene	1,055	1,057–1,059^a^ ^,^ ^b^	0.2
9	2-Nonanone	1,095	1,090–1,096^a^ ^,^ ^b^	0.2
10	α-Campholenal	1,120	1,122–1,126^a^ ^,^ ^b^	0.4
11	*cis-*Limonene oxide	1,129	1,132^a^	0.2
12	*trans*-Pinocamphone	1160	1159–1162^a^ ^,^ ^b^	29.5
13	Pinocarvone	1,162	1,164–1,165^a^ ^,^ ^b^ ^,^ ^c^	21.8
14	*cis*-Pinocamphone	1169	1174–1175^b^ ^,^ ^c^	8.0
15	Cumenol	1,196	1,196–1,198^a^ ^,^ ^b^	0.1
16	Carvone	1,228	1,231^a^	0.1
17	3-Cyclohexen-1-one-2 isopropyl 5 methyl	1,273	1,274^a^	0.2
18	Ethanone,1-(2-hydroxy-5-methylphenyl)	1,277	1,276^d^	0.1
	Oil yield (%)		0.5	
	Total identified (%)		93.9	
	Grouped compounds (%)			
	Monoterpene hydrocarbons		31.8	
	Oxygenated monoterpenoids		62.1	

a NIST 14.

b Adams, 2007.

c García-Roja et al., 2009.

d Lei et al., 2018.

The constituent compounds are listed in the order of elution from an HP-5MS column using a homologous series of C8–C20 n-alkanes. RI exp: Experimental Retention Index of an HP-5MS column by co-injection of a homologous series of n-alkanes C8–C20 and determination of the respective linear retention index. RI lit: Literature Retention Index. Method of identification: MS, by comparing the mass spectrum with those in the digital mass libraries NIST 14 and Adams, 2007.

### Antimicrobial activity

The antifungal activity of the essential oil of *A. porophyllum* var. *cancellatum* was demonstrated by inhibition of the growth of *C. glabrata* (10 ± 0.0 inhibition zone), *C. albicans* (20 ± 0.0 inhibition zone), and *C. tropicalis* (15 ± 0.0 inhibition zone)*,* all of which were isolated from clinical cases. *C. glabrata* was determined to be the most sensitive species to the essential oil, with an MIC of <0.06 mg/ml compared to the 0.125 and 0.250 mg/ml for the other species, respectively. Furthermore, the essential oil inhibited the growth of ten bacterial strains, five gram-positive, and five gram-negative strains. The most sensitive bacterial strains were *S. aureus* cc (9.33 ± 0.6 inhibition zone; MIC 1 mg/ml), *E. coli* 82MR (8.00 ± 0.0 inhibition zone; MIC 0.75 mg/ml), and *K. pneumoniae* (16.00 ± 0.0 inhibition zone; MIC 0.75 mg/ml) (Table 2). The essential oil substantially prevented the formation of biofilms in *C. albicans*, *S. aureus* cc, and *E. coli* 82MR by 46.3%, 47.6%, and 33.1%, respectively, with respect to the untreated control (Table 3).

**TABLE 2:** Anti-*Candida* and antibacterial activities of the essential oil of *A. porophyllum* var. *cancellatum*.

Yeast strain	*A. porophyllum* var. *cancellatum*			Positive control: Nystatin	
	Inhibition zone (mm)^a^	MIC (mg/ml)	MFC (mg/ml)	Inhibition zone (mm)^b^	MIC (μg/ml)
*C. albicans* 17MR	20 ± 0.00	0.125	0.25	30.00 ± 0.81	11.00
*C. glabrata*	10 ± 0.00	<0.06	—	20 ± 0.00	8.00
*C. tropicalis*	15 ± 0.00	0.25	0.5	20.33 ± 0.47	9.00

Bacteria strains	*A. porophyllum* var. *cancellatum*			Positive control: Chloramphenicol	
	Inhibition zone (mm)	MIC (mg/ml)	MBC (mg/ml)	Inhibition zone (mm)^c^	MIC (μg/ml)
*M. luteus* ATCC 10204	9.67 ± 0.58	2.00	3.00	23 ± 0.50	2.00
*S. aureus* ATCC 29213	10.00 ± 0.00	2.00	3.00	27.6 ± 0.10	8.00
*S. aureus* cc	9.33 ± 0.58	1.00	2.00	14.00 ± 0.00	2.00
*S. aureus* CUSI	7.00 ± 0.00	2.00	3.00	25.00 ± 0.00	4.00
*S. epidermidis* FES-C	10.33 ± 0.58	>3.00	—	6.70 ± 1.20	4.00
*E. coli* 82MR	8.00 ± 0.00	0.75	1.00	30.66 ± 0.94	4.00
*E. coli* CUSI	6.00 ± 0.00	2.00	3.00	22.33 ± 0.47	4.00
*K. oxytoca* ATCC 8724	10.67 ± 0.58	2.00	3.00	21.33 ± 0.48	1.00
*K. pneumoniae* ATCC 13883	16.00 ± 0.00	0.75	1.00	12.33 ± 0.94	1.00
*S. marcescens* ATCC 14756	6.00 ± 0.00	>3.00	—	20.00 ± 0.00	2.00

a 3.25 mg/per disk.

b 50 μg/ml.

c 25 μg/ml.

Average of three repetitions ± standard deviation; --undetermined. MIC, minimum inhibitory concentration; MFC, minimum fungicidal concentration; MBC, minimum bactericidal concentration.

**TABLE 3:** Antibiofilm activity of the essential oil of *A. porophyllum* var. *cancellatum*.

Strain	Concentration (mg/ml)	% Antibiofilm activity
*C. albicans* 17MR	MIC 0.125	32.7 ± 12.02
	MFC 0.250	46.3 ± 6.81
*S. aureus* cc	MIC 1.00	42.2 ± 7.17
	MBC 2.00	47.6 ± 6.60
*E. coli* 82MR	MIC 0.750	29.9 ± 14.54
	MBC 1.00	33.1 ± 11.47

Average of eight repetitions ± standard deviation. MIC, minimum inhibitory concentration; MFC, minimum fungicidal concentration; MBC, minimum bactericidal concentration.

### Anatomical study of the leaf

According to Villarreal (2003), *A. porophyllum* var. *cancellatum* is an annual herbaceous plant with oval oil glands in its leaves (Figure 1B,C) and floral involucre bracts. Leaves were 2–6 cm long, pinnatisect (7–13 lobes linear to obovate), with the lobes toothed to split, and some teeth ending in long bristles (Figure 1B). The leaf anatomy of the species is shown in Figure 1D–N. In the superficial view of the leaf epidermis, the epidermal cells had undulated anticlinal walls, anisocytic and a few anomocytic stomata, (Figure 1D). The main types of trichomes were as follows: 1) uniseriate simple with a foot formed by 1–2 rounded thick-walled basal cells, and a head containing up to seven thin-walled cells, some of which showed collapsed walls or contents, frequently oriented parallel to the epidermal surface. In some cases, trichomes were observed with a flexuous, wavy, and ribbon-like appearance (Figure 1E,F); 2) glandular with a uniseriate foot formed by 1–4 cells and a head consisting of 2–5 irregularly shaped or rounded cells (Figures 1G–I); 3) glandular with a uniseriate foot made up of up to four cells and a club-shaped or rounded head, formed by four rows of cells with dense cytoplasm; these were scarce (Figure 1J).

In the transverse view (Figure 1K,L), the leaves were amphistomatic. The cuticle was smooth, with a simple epidermis; bifacial mesophyll, with uni-bistratified palisade parenchyma and compact spongy with 5–6 strata of quadrangular to rounded cells; collateral vascular bundles, those of the second level (secondary veins) surrounded by a parenchymal sheath (Figure 1K). The middle vein had a triangular outline with protuberances toward both surfaces, the lower one more prominent and thinner toward the underside; trichomes were scarce and of the same type as those described for the rest of the leaf blade; cuticle was crenate, with cells of the abaxial epidermis more voluminous than those of the rest of the lamina. Parenchymatous or collenchymatous cells with slightly thickened walls were observed under the adaxial and abaxial epidermis. These walls were thinner toward the center of the midvein, where they surrounded the only collateral-type vascular bundle, which was circular and encased in a parenchyma sheath. The palisade and spongy parenchyma penetrate part of the midvein (Figure 1L).

In the narrowest areas of the leaf blade, two large glands, approximately 1–2 mm long, were located on each side of the midvein (Figure 1C,M). In the transverse view, the cuticle was a smooth, simple epidermis, with rounded dome-like cells on both surfaces; below which there were 4–5 layers of tangentially elongated cells with thickened walls and narrow lumens surrounding the glands. The glands were elliptical in shape and almost occupied the entire mesophyll (Figure 1N). They were of lysigenous origin; each gland was associated with a pair of vascular bundles at its ends. Toward the margin of the lamina there was chlorenchyma, which sometimes partially surrounded each gland and extended into the midvein. The cuticle was crenate below which there were several layers of collenchyma associated with the central and semicircular collateral vascular bundle.

## Discussion

*A. porophyllum* var. *cancellatum* is one of the 12 species of the genus *Adenophyllum* distributed in the southwestern United States, Mexico, Central America, and the Antilles (Villarreal, 2003). Phytochemical studies on the genus *Adenophyllum* indicate that thiophenes are a constituent compound of this herb in organic extracts, with terthiophenes being the most abundant (Downum et al., 1985). Hexanedioic acid and bis (2-ethylhexyl) ester are the main component of the ethyl acetate extract of *A. porophyllum* and exhibits activity against the phytopathogenic fungi, *Pestalotiopsis clavispora*, *Colletotrichum gloeosporioides,* and *Lasiodiplodia pseudotheobromae* (Hernández-Ceja et al., 2021). In addition, methanolic and ethyl acetate extracts of the roots of *A. aurantium* have antifungal activity against the phytopathogenic mycelial fungi, *Alternaria alternata* and *Fusarium solani* (Lira-De León et al., 2014). For other species of the genus antifungal activity are reported but no its antibacterial activity as well as their essential oils.

The essential oil of *A. porophyllum* var. *cancellatum* exhibits broad-spectrum antimicrobial activity, inhibiting the growth of both yeasts and bacteria. *Candida* species are the most sensitive to this oil. To the best of our knowledge, this is the first report on the chemical composition and antimicrobial potential of the essential oil, and the anatomy of the plant.

*A. porophyllum* var. *cancellatum* produces mainly monoterpenoids, the most abundant are *trans*-pinocamphone, limonene, pinocarvone, and *cis*-pinocamphone, which make up 84% of the essential oil. There are no studies on the essential oils from the other species of the genus *Adenophyllum* for comparison; however, it is known that other species in the Tageteae tribe, such as *Dyssodia acerosa* (Tellez et al., 1997), *D. tagetiflora* (García-Bores et al., 2018), *D. decipiens* (Pacheco-Hernández et al., 2020), and several species of the genus *Tagetes* (Ali et al., 2014) mainly produce monoterpenoids. In all of them, limonene is one of the main components of the essential oils.

There are species that mainly produce pinocamphone and pinocarvone in essential oils. In *Hyssopus officinalis* (Lamiaceae), *cis*-pinocamphone, *trans*-pinocamphone, pinocarvone, pinene, and phellandrene are the most abundant constituents; there is quantitative variability in the chromatographic profiles depending on variety, genotype, region, and culture conditions (Chalchat et al., 1999; Fraternale et al., 2004; Hristova et al., 2015; Stappen et al., 2015; Sharifi-Rad et al., 2022). The composition of hyssop oil is linked to its medicinal properties. The plant is traditionally used for its antiseptic properties in the treatment of infectious diseases, chronic bronchitis, and asthma (Stankovic' et al., 2016). The essential oil of *H. officinalis* has antibacterial activity, with an MIC of 4,000 μg/ml against *S. aureus* and 2000 μg/ml against *E. coli* (Stappen et al., 2015). In addition, the oil exhibits antifungal activity against *Fusarium* (Fraternale et al., 2004) and *Candida* (Hristova et al., 2015) strains.

The essential oil from the aerial parts of *Cedronella canariensis* var. *canariensis* (Lamiaceae) contains mainly pinocarvone, 46.8%–58.0% (López-García et al., 1992; Zorzetto et al., 2015). The oil inhibits the growth of Gram-positive and Gram-negative bacteria; however, it is more active against fungi of the genera *Candida*, *Cryptococcus*, and *Saccharomyces*, with zones of inhibition between 46 mm and 60 mm (López-García et al., 1992). The essential oil of *Myrothamnus moschatus* (Myrothamnaceae) mainly contains oxygenated monoterpenoids, *trans-*pinocarveol (35.6%), and pinocarvone (20.0%); coincidentally, the essential oil of this species exhibits antimicrobial activity against *C. albican*s (with an inhibition zone diameter of 18 mm) (Nicoletti et al., 2012). However, the antimicrobial activity of pure pinocarvone remains unknown.

Pinocamphones (*cis* and *trans*) and limonene are the main monoterpenoids in the essential oil of *A. porophyllum* var. *cancellatum*. The presence and quantity of these three monoterpenoids could be responsible for the antimicrobial activity of the essential oil of the plant because they constitute around 59.3% of the essential oil. Hristova et al. (2015) studied the anti-*Candida* activities of *trans*-pinocamphone and *cis*-pinocamphone. Both isomers have MICs of 1,000 μg/ml, indicating that isomerization does not influence activity. Thakre et al. (2018) reported that limonene exhibits antimicrobial activity against *C. albicans* and inhibits planktonic growth (yeast), morphogenesis (hyphae), and biofilm growth. Furthermore, the application of this compound resulted in a concentration-dependent reduction in the biofilm formation by several species of *Streptococcus* (Subramenium et al., 2015). In cells of the yeast, *Zygosaccharomyces rouxii*, limonene destroys yeast proteins, inhibits their synthesis, produces nucleic acid leakage, and alters membrane permeability (Cai et al., 2019). In *E. coli*, (-)-limonene acts by producing oxidative stress through the formation of significant amounts of hydrogen peroxide and superoxide anion radicals, which damage DNA and the cell membrane, increase permeability, resulting in cell death (Melkina et al., 2021).

The antimicrobial activity of essential oils is generally attributed to their major components, in part because it is difficult to discriminate the biological activities of minor compounds. Oxygenated terpenoids with groups aldehydes, and alcohols are the most active, followed by those containing esters and ketones, while hydrocarbons are generally ineffective. However, there is a synergistic effect between compounds with strong, moderate, and low antimicrobial activities (Nikkhah et al., 2017; Pinto et al., 2020). Hydrocarbon-type terpenoids could facilitate the transmembrane transport of oxygenates, resulting in a synergistic effect on antimicrobial activity (Nguefack et al., 2009). This could explain why essential oils are more active than pure compounds. For instance, the *H. officinalis* oil is more active in several species of *Candida* than its main components *cis*- and *trans*-pinocamphone, α- and β-pinene, and β-phellandrene. The MICs values of the pinocamphones were 28% and 21% higher than that of hyssop oil, against *C. albicans* and *C. glabrata*, respectively (Hristova et al., 2015). In the case of *A. porophyllum* var. *cancellatum* essential oil, it is possible that the hydrocarbon-type monoterpenes, such as limonene, have a synergistic effect with pinocamphones. However, further studies are needed to verify this hypothesis as well as the mechanism of action.

The anatomical characteristics of other *Adenophyllum* species have not yet been recorded. Therefore, as part of the pharmacognosy investigation of *A*. *porophyllum* var. *cancellatum*, the leaf microstructure was compared with that in the other taxa of the Tageteae tribe (Supplementary Table S1), because there are shared characteristics, such as amphiestomatic leaves, anisocytic/anomocytic stomata, and parenchyma sheath surrounding veins. Parenchyma sheaths are associated with Kranz-type photosynthesis, which appears to occur in dry climates, such as tropical deciduous forests, scrubs, grasslands, and disturbed areas where this species grows (Villarreal, 2003). Kranz-type anatomy is reported in different Asteraceae (Sánchez et al., 1986; Peter and Katinas, 2003). The anatomical characters are more homogeneous within the Tageteae tribe than at the level of the Asteraceae family (Rivera et al., 2017). Nevertheless, trichomes can contribute to the delimitation of species or genera belonging to the Tageteae tribe (Milan et al., 2006; Azevedo, 2007; García-Sánchez et al., 2012; Ferraro and Scremin-Dias, 2018; Rivera et al., 2019; Younis et al., 2020). In addition, a single central vascular bundle in the midvein and the size and shape of the glands could differentiate *A. porphyllum* var. *cancellatum* from the other closely related taxa.

Considering the high essential oil production of this taxon, further comparative analyzes of chemistry, antimicrobial activity, and leaf anatomy between different *Adenophyllum* species are needed.

## Conclusion

This study validated the medicinal use of *A*. *porophyllum* var. *cancellatum* for the treatment of infections. The essential oil from its aerial parts showed antimicrobial properties against bacteria and yeasts of medical importance. The main chemical components of the oil were *trans*- and *cis*-pinocamphone, limonene, and pinocarvone, which could be responsible for the activity and could potentially be chemical markers of the species. An anatomical study of the leaf enabled characterization of the oil-producing glands and other histological attributes to support the identification of this species for quality control purposes.

## Data Availability

The original contributions presented in the study are included in the article/Supplementary Material, further inquiries can be directed to the corresponding author.
