# Supplementary material for: Chemical profile, antimicrobial activity, and leaf anatomy of Adenophyllum porophyllum var. cancellatum
Source: Front Pharmacol. 2022 Oct 11;13:981959. doi: 10.3389/fphar.2022.981959 (PMC9592750; doi:10.3389/fphar.2022.981959)
Supplement: Supplementary file 1 [file DataSheet2.PDF]

Species / Characteristics	*Adenophyllum porophyllum* var. *cancellatum* (Cass.) Strother	*Tagetes* spp.[A,G,R,Y]	*Dyssodia papposa* (Venth.) Hitchc.[R]	*Thymophylla tenuiloba* Small[Y]	*Pectis prostrata* Sieber ex Less.[R] *P. gardneri* Baker[F] *P. brevipedunculata* Sch.Bip[SO]	*Porophyllum ruderale* M.Gómez[M,R]
Trichomes	si (uni), gl (head consisting of 2-5 cells irregularly shaped or rounded club-shaped trichome	abs; si (co, tu, as, sp, sup, p); gl (ca, uni, co).	abs	ca mushroom-like	abs; si, uni	abs
Cuticle	smooth	striate or smooth	striated or smooth	---	striated or smooth	ornamented
Stomata	anisocytic, anomocytic scare	anomocytic, anisocytic scare,	anomocytic or anisocytic	anomocytic	anomocytic or anisocytic	anomocytic, few anisocyti
Leaf	amph	amph	amph	amph	amph	amph
Number of vascular bundles in middle vein	1	diverse number	1	---	3	1
Bundle sheath	+	+	+	---	+	not described
Secretory cavities	crescent-shaped glands with rounded ends length 2 mm	*T. erecta*: rounded schizogenic channels	canals associated with vascular bundles or in the mesophyll	oval	canals associated with vascular bundles or in the mesophyll/ schyzolysigenous secretory cavities	secretory cavities originated by cell lysis

Supplementary table 1. Leaf microstructure compared with other taxa of the Tageteae tribe. abs= absent; gl= glandular; ca= capitated; si= simple; gl= glandular; co= conical; tu= tubular; as= apex sagittate; p= papille; uni= uniseriate; sp= spheroid; sup= subspheroidal; += present; [A]= Anaya-Gutiérrez et al. (2022); [G]= García-Sánchez et al. (2012); [R]= Rivera et al. (2019); [Y]= Younis et al. (2020); [F]= Ferraro and Scremin-Dias (2018); [SO]= Azevedo (2007); [M]= Milan et al. (2006).
