# Supplementary material for: Chemical profile, antimicrobial activity, and leaf anatomy of Adenophyllum porophyllum var. cancellatum
Source: Front Pharmacol. 2022 Oct 11;13:981959. doi: 10.3389/fphar.2022.981959 (PMC9592750; doi:10.3389/fphar.2022.981959)
Supplement: Supplementary file 2 [file DataSheet1.PDF]

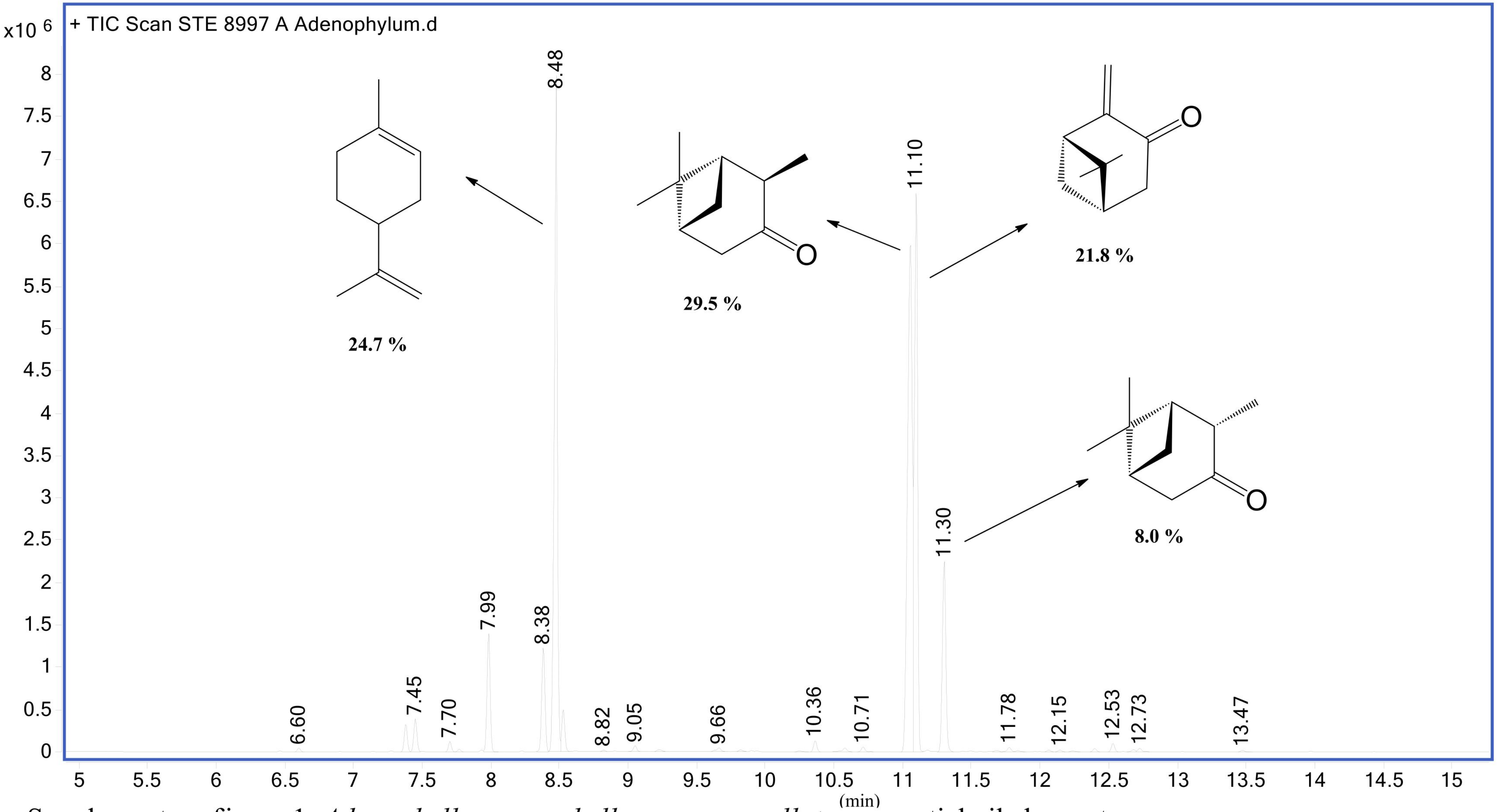

Supplementary figure 1. *Adenophyllum porophyllum* var. *cancellatum* essential oil chromatogram
